# CRX-527 induced differentiation of HSCs protecting the intestinal epithelium from radiation damage

**DOI:** 10.3389/fimmu.2022.927213

**Published:** 2022-08-30

**Authors:** Dongshu Liu, Suhe Dong, Cong Liu, Jicong Du, Sinian Wang, Huijie Yu, Wei Li, Zhongmin Chen, Renjun Peng, Qisheng Jiang, Mengying Zou, Fengsheng Li, Rong Zhang

**Affiliations:** ^1^ Postgraduate Training Base of the People's Liberation Army (PLA) of China Rocket Force Characteristic Medical Center, Jinzhou Medical University, Beijing, China; ^2^ People's Liberation Army (PLA) of China Rocket Force Characteristic Medical Center, Beijing, China; ^3^ Naval Medical University, Shanghai, China

**Keywords:** irradiation damage, hematopoiesis, intestinal stem cells, macrophage activation, TLR4 activation

## Abstract

Recently, Toll-like receptors (TLRs) have been extensively studied in radiation damage, but the inherent defects of high toxicity and low efficacy of most TLR ligands limit their further clinical transformation. CRX-527, as a TLR4 ligand, has rarely been reported to protect against radiation. We demonstrated that CRX-527 was safer than LPS at the same dose *in vivo* and had almost no toxic effect *in vitro*. Administration of CRX-527 improved the survival rate of total body irradiation (TBI) to 100% in wild-type mice but not in TLR4^-/-^ mice. After TBI, hematopoietic system damage was significantly alleviated, and the recovery period was accelerated in CRX-527-treated mice. Moreover, CRX-527 induced differentiation of HSCs and the stimulation of CRX-527 significantly increased the proportion and number of LSK cells and promoted their differentiation into macrophages, activating immune defense. Furthermore, we proposed an immune defense role for hematopoietic differentiation in the protection against intestinal radiation damage, and confirmed that macrophages invaded the intestines through peripheral blood to protect them from radiation damage. Meanwhile, CRX-527 maintained intestinal function and homeostasis, promoted the regeneration of intestinal stem cells, and protected intestinal injury from lethal dose irradiation. Furthermore, After the use of mice, we found that CRX-527 had no significant protective effect on the hematopoietic and intestinal systems of irradiated TLR4-/- mice. in conclusion, CRX-527 induced differentiation of HSCs protecting the intestinal epithelium from radiation damage.

## Graphical Abstract

CRX-527 induced differentiation of HSCs protecting the intestinal epithelium from radiation damage. To evaluate the radiation protection effect of CRX-527 on mice irradiated with lethal dose, CRX-527 was intraperitoneally injected with 0.5 mg/kg 24 hours and 2 hours before TBI, respectively. CRX-527 increased the proportion and number of hematopoietic stem cells, stimulated the differentiation of hematopoietic stem cells into macrophages, and activated the TLR4 pathway of macrophages. Macrophages activation protected the inherent cells of the intestines from radiation damage after irradiation, promoted the repair of intestinal structure and function, inhibited apoptosis and pyroptosis, and alleviated DNA damage caused by ionizing radiation. CRX-527 may be a potential radiation protection agent.

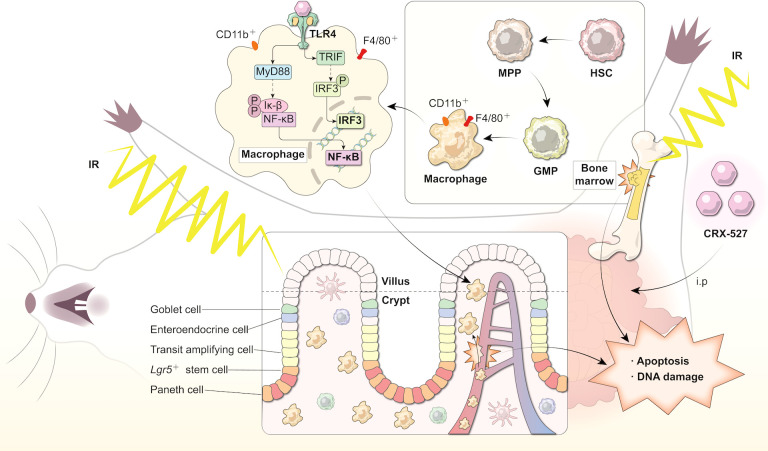

## 1 Introduction

The rapid development of nuclear science and technology has greatly benefitted human beings, but it has also brought radiation damage caused by nuclear explosion or nuclear leakage, which can harm many tissues and organs. These serious irradiation exposures may lead to acute radiation syndrome, resulting in multiple organ damage, including damage to the hematopoietic system, digestive system and nervous system, and even death (1).

Death caused by irradiation is usually due to poor hematopoietic recovery or depletion of the intestinal epithelium. The effect of irradiation on hematopoiesis, namely, hematopoietic acute radiation sickness, is mainly manifested in injury to the hematopoietic system, including the bone marrow and spleen (2). Because of the high sensitivity of hematopoietic stem cells (HSCs) to ionizing radiation, low-dose ionizing radiation can even affect their proliferation and differentiation (3). Meanwhile, the effect of irradiation on the intestine is another subtype of acute ionizing radiation injury, which usually occurs in the process of high-dose ionizing radiation, defined as gastrointestinal acute radiation sickness. In the digestive system, the small intestine has high radiation sensitivity, which can lead to the fracture and shedding of villi, loss of crypts, inflammation, exudation, hydration, and increase of intestinal permeability (4).

Immune defense cells differentiated from the hematopoietic system, such as macrophages and monocytes, play an important role in immune regulation. The digestive system, as an open system, is particularly important in immune defense, and macrophages are essential for intestinal homeostasis and intestinal physiology (5). In the process of ionizing radiation, the collapse of the immune system may be a crucial pathological process in the occurrence and development of gastrointestinal acute radiation sickness. Therefore, mediating intestinal immune function may balance radiation prevention and treatment (6). Some scholars found that the TLR4 receptor agonist MPLA could protect mouse spermatogonia from radiation damage by activating TLR4 pathways in macrophages, which suggested that stimulating immune defense may resist ionizing radiation damage to multiple tissues and organs (7). It has not yet been determined whether enhanced immune responses mediated by HSCs benefit intestinal homeostasis after irradiation through the increased proportion of macrophages and monocytes (8).

Toll-like receptors (TLRs) have been verified to protect against radiation damage, and the TLR5 agonist CBLB502 was first developed as an effective radioprotective agent (9). From the same gene families, TLR4 can mediate innate immunity and participate in radiation protection (10). TLR4 and TLR5 are both important components of toll-like receptors (TLRs), which play an important role in natural immunity. However, TLR5 ligands are mainly bacterial flagellin, while TLR4 ligands are mainly LPS analogs. CRX-527 is a synthetic lipid A belonging to the amino acylglucosamine 4-phosphate (AGP) family (11), and is the active ingredient of lipopolysaccharide (LPS). Similar to LPS, CRX-527 can also activate TLR4 (12). Because LPS can activate TLR4, and LPS has been reported to have a radiation protection effect, but its toxicity is relatively large, and it is not easy to be used in clinical practice, CRX-527 was developed as a vaccine adjuvant, its safety is much higher than LPS, and it induces hematopoietic stem cells to differentiate into macrophages by activating the hematopoietic system. The mechanisms that protect the gut from radiation damage have opened up new avenues of research. Our research confirmed that CRX-527 could protect the hematopoietic system from ionizing radiation damage and promote the proliferation and differentiation of HSCs to macrophages, thus protecting the intestinal system from radiation damage. This research is a pioneering work.

### 1.1 Research in context

#### 1.1.1 Evidence before this study

Toll-like receptors (TLRs) have been verified to protect against radiation damage. The TLR5 agonist CBLB502 was first developed as an effective radioprotective agent. From the same gene families, TLR4 can mediate innate immunity and participate in radiation protection. CRX-527 is a synthetic lipid A belonging to the amino acylglucosamine 4-phosphate (AGP) family, and is the active ingredient of lipopolysaccharide (LPS). Similar to LPS, CRX-527 can also activate TLR4. Our research confirmed that CRX-527 could protect the hematopoietic system from ionizing radiation damage and promote the proliferation and differentiation of HSCs to macrophages, thus protecting the intestinal system from radiation damage. This research is a pioneering work.

#### 1.1.2 Added value of this study

Nowadays, radiation protective agents and key molecules in radiation damage have become the focus of research. Bone marrow radiation sickness and intestinal radiation sickness are the two main types of acute radiation sickness. Currently, there are no other timely and effective prevention and treatment methods except WR-2721 approved by FDA in the United States. On the basis of previous studies on TLR-related pathways, this study extends outward to verify the mechanism of TLR4 pathway against radiation by CRX-527, a new agent with incomplete anti-radiation mechanism, to explore the role of CRX-527 in the protection of ionizing radiation damage in hematopoietic system and intestines, and to preliminarily reveal how CRX-527 activates the immune system to protect against intestinal damage.

#### 1.1.3 Implications of all the available evidence

The aims of this study were (1) CRX-527 protected against irradiation-induced hematopoietic damage and promoted immune-activated differentiation of hematopoietic stem cells. (2) CRX-527 promoted the preservation and regeneration of ISCs and prevented radiation-induced intestinal injury. (3) Activation of TLR4 mediated the protective effect of CRX-527 on hematopoietic and intestinal radiation injury.

## 2 Methods

### 2.1 Animals and cells

C57BL/6 mice were purchased from SPF (Beijing) Biotechnology Co., Ltd. Homozygous TLR4 knockout mice (TLR4^-/-^) were purchased from Southern Model Animal Co., Ltd. (Shanghai, China), all of which were raised in the intelligent IVC system of the animal room of the Rocket Force Characteristic Medical Center. All experimental procedures were carried out in accordance with the Guidelines for the Care and Use of Laboratory Animals (8th Edition, National Academy of Sciences Press, Washington, D.C., 2011) and approved by the ethics review committee. Mouse intestinal epithelial cells (MODE-K), mouse macrophages (RAW264.7), human small intestinal epithelial cells (HIECs), and human mononuclear macrophages (THP-1) were purchased from Eallbio Life Sciences (Beijing, China). RAW264.7 cells were cultured in DMEM, and the other three cells were cultured in RPMI 1640 medium containing 10% fetal bovine serum (Gibco, USA), 100 U/ml penicillin and 100 mg/ml streptomycin (Gibco, USA). The cells were cultured in an incubator at 37°C and 5% CO_2_.

### 2.2 Irradiation and treatment

HIECs and MODE-K cells were irradiated with a single dose of 16 Gy X-ray using an irradiator (KUBTEC XCELL 225, 225 KV 13.2 mA 1 Gy/min), while unirradiated control cells were studied in parallel under the same conditions. For mice, 5 Gy total body dose was used to observe the changes of hematopoietic system, 7.5 Gy total body dose was used to observe the changes of intestinal system, and 9 Gy local abdominal irradiation was used to observe the changes of intestinal system. Mice received 0.5 mg/kg CRX-527 by intraperitoneal injection 24 hours and 2 hours before irradiation. Whole body irradiation mice were fixed with a fixed frame and then placed in an irradiator for irradiation. After irradiation, the corresponding tissues were collected for detection.

### 2.3 Survival analysis

C57BL/6 mice in the same batch weighing 18-20 g were selected as the control group (n=10) and CRX-527 administration group (n=10). According to different experimental purposes, mice were given different doses of single whole body X-ray irradiation. The survival of mice was recorded for 30 consecutive days, and a survival curve was drawn.

### 2.4 Peripheral blood cells assay

The peripheral blood of mice was detected by a veterinary blood cell three-classification counter (URIT-5160Vet, China). A capillary glass tube was inserted into the inner canthus vein of mice. Two to three drops of blood were taken into an anticoagulant tube containing heparin, quickly mixed and counted.

### 2.5 Mouse spleen coefficient assay

The spleen coefficient refers to the ratio of the weight of the spleen to the body weight of mice. Mice were given a single dose of 5 Gy of wholebody irradiation. Body weight and spleen were measured at 0, 1, 7, 14 and 30 days to calculate the spleen coefficient.

### 2.6 Flow cytometric assay of HSCs

The bone marrow cells (suspension) were diluted to an appropriate concentration; flow cytometry antibody was added, and the cells were fully mixed. Cells were then incubated for 20-30 min in the dark at room temperature. PBS was added to the tube, diluted, mixed well, and detected on an analyzer.

### 2.7 H&E staining and immunohistochemistry (IHC)

The intestine, spleen and femur were fixed in 4% paraformaldehyde solution for 24 hours and washed repeatedly. The femur was incubated with decalcification solution for 24 hours, dehydrated with alcohol, cleared by xylene I and II solutions, embedded and sliced by a slicer with a thickness of 3 µm, baked and dewaxed. The slides were stained with hematoxylin and eosin, dehydrated with absolute ethanol and sealed. H&E staining was performed until hydration, followed by antigen repair, rinsing, peroxidase blocking, and blocking. The blocking solution was discarded, 50 µl of primary antibody (*Ki67, Zo-1, 8-OHdG, Lysozyme, Villin*) was added dropwise, and the cells were incubated at 4°C overnight. The sections were rinsed and incubated with secondary antibody at room temperature for 1 h and stained with DAB. Microscopic examination was used to evaluate the degree of staining.

### 2.8 Western blotting assay

Intestines or cells were lysed with RIPA lysate. The protein concentration was determined by a BCA protein detection kit (Biyuntian, China). Thirty grams of total protein was separated by 10% SDS–PAGE and transferred to a polyvinylidene fluoride membrane (Bio–Rad, USA). The membrane was blocked with 5% skim milk for 1 hour and then incubated with antibody at 4°C overnight. After three washes with TBST (10 minutes each), a secondary antibody (1:50,000, Abcam, UK) was incubated for 1 hour. After chemiluminescence, Image J software was used to quantify the gray values of the target band and internal reference (GAPDH).

### 2.9 Macrophage assay

Dilution of bone marrow cells (suspension) to appropriate concentration; Flow cytometry antibody labeled macrophages (F4/80+,CD11b+) were added and the cells were fully mixed. Incubate at room temperature in darkness for 20-30 minutes. PBS was added into the test tube, diluted, mixed evenly, and detected by an analyzer.

### 2.10 Co-culture assay

In brief, 1×10^5^ RAW264.7 or THP-1 cells were seeded in the Transwell chamber (BIOFIL, 0.4 µm, 6.5 mm diameter), while MODE-K or HIECs were seeded at the bottom of the 12-well plates and then cultured according to the manufacturer’s instructions. RAW264.7 cells or THP-1 cells in the Transwell chamber were treated with CRX-527 12 hours before irradiation. After irradiation, MODE-K cells or HIECs on 12-well plates were used for colony formation, ROS, and Western blot analysis.

### 2.11 Clone formation assay

The cells were inoculated in 60 mm culture dishes. Different irradiation doses of 0, 2, 4, 6, and 8 Gy were seeded at densities of 500, 1000, 4000, 8000, and 16000 cells, respectively. Continuous culture was performed for 10-15 days, and the solution was changed every three days. The cells were photographed under a microscope, and the clone formation fraction was calculated.

### 2.12 ROS assay

ROS levels in MODE-K cells and HIECs were detected according to the manufacturer’s instructions using an ROS detection kit (Gene Copoeia, A507). The generation of ROS was detected by flow cytometry at 495/529 nm.

### 2.13*In situ* hybridization

The tissue was removed and cleaned and immediately placed in fixative solution (DEPC water) for fixation for more than 12h. After the fixation, the tissue was dehydrated by gradient alcohol, dipped in wax and embedded. The paraffin was sliced by the slicer, the slices were collected by the spreading machine and baked in a 62° oven for 2h.The slices were placed in dewaxing transparent solution i 15min-dewaxing transparent solution ii 15min-anhydrous ethanol i 5min-anhydrous ethanol ii 5min, then dried naturally and soaked in DEPC water. According to the duration of tissue fixation, the slices were boiled in repair solution for 10-15 minutes and cooled naturally. After gene circle, according to the characteristics of different indicators of different tissues, add protease K(20ug/mL) for 37° digestion min. After rinsing with pure water, wash with PBS 3 times ×5min. The pre-hybridization solution was dropped and incubated at 37°C for 1h. Pour the pre-hybridization solution, drop the hybridization solution containing probe, concentration, incubator hybridization overnight. Wash off the hybrid solution, 2×SSC, 37°C for 10min, 1×SSC, 37°C for 2×5min, 0.5×SSC, room temperature for 10min. Formamide washing can be added if there are many nonspecific hybrids. Section drops were added with DAPI dye solution, and incubated for 8min away from light. After rinsing, anti-fluorescence quenching sealing tablets were added to seal the slices. Sections were observed under nikon positive fluorescence microscope and images were collected.

### 2.14 Positive statistics

The length of villi in the small intestine was measured under the microscope for Villin data statistics. The immunofluorescence intensity of 8-OHDG and the staining intensity of ZO-1 were measured by Image J.

### 2.15 Statistical analysis

The data were analyzed by GraphPad Prism 7 (GraphPad software, San Diego, Calif., US) and expressed as the mean ± mean standard error (SEM). One-way ANOVA or two-way ANOVA was used to analyze the data. All experiments were repeated at least three times. *P < 0.05 indicated statistical significance.

## 3 Results

### 3.1 CRX-527 protected against hematopoietic injury induced by ionizing radiation

**Figure 1:**
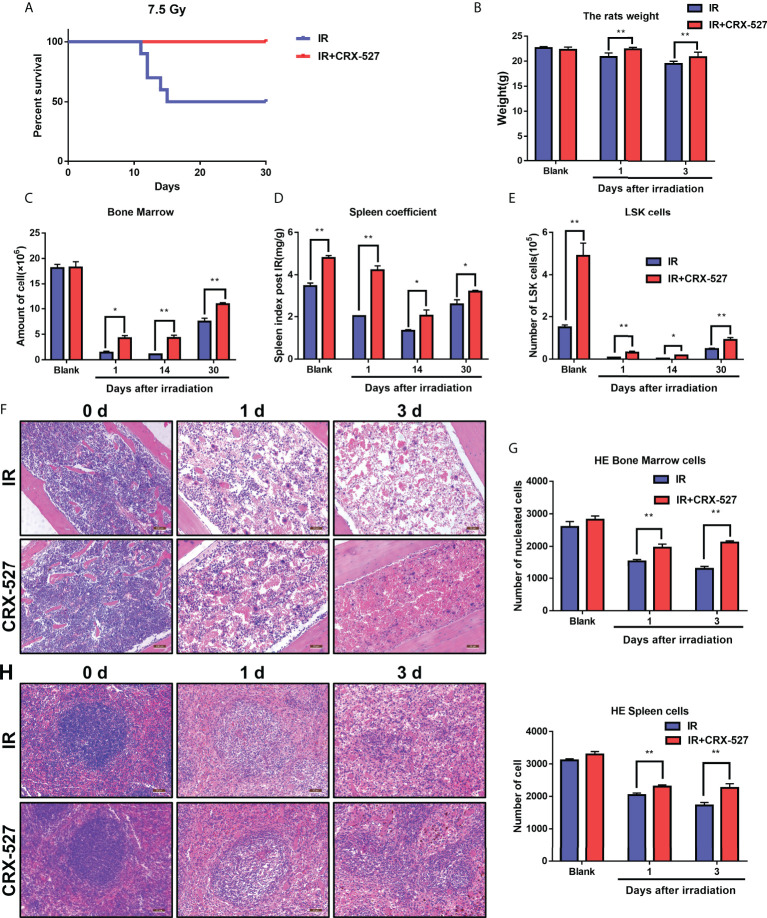
CRX-527 protected against hematopoietic injury induced by ionizing radiation. **(A)** Mice survival curve of 7.5 Gy total body irradiation. **(B)** Body weight changes in 7.5 Gy irradiated mice at different times. **(C)** Changes in spleen coefficients in mice at different times. **(D)** Nucleated cell counts in the bone marrow of mice at different times. **(E)** The nucleated cell count in the bone marrow of mice at different times. **(F)** H&E staining of bone marrow of irradiated mice. **(G)** Statistics of nucleated cells in bone marrow of H&E-stained mice. **(H)** H&E staining of spleens of irradiated mice. **(I)** Statistics of nucleated cells in spleens using H&E analysis. The data are expressed as the mean ± SEM, *p < 0.05, **p < 0.01.

First, we clarified the protective effect of CRX-527 on hematopoietic injury induced by ionizing radiation. The survival rate of mice in the irradiation group was 50% under a lethal dose of 7.5 Gy, while that of the CRX-527 group was 100%, which indicated that CRX-527 could significantly improve the survival rate of mice after irradiation (**Figure 1A**). Moreover, the safety of CRX-527 was significantly better than LPS at the same dose (**Figure S1**). Meanwhile, we recorded the weight changes of mice and found that the weight changes of CRX-527-treated mice were more gradual, while the weight of IR mice was sharply reduced (**Figure 1B**). We found that spleen volume also increased after CRX-527 treatment (**Figure S1B**). Furthermore, we detected the related indices of the hematopoietic system involved in radiation damage. After IR, CRX-527-treated mice maintained more nucleated cells in bone marrow, while those in irradiated mice were almost exhausted (**Figure 1C**). The change in spleen coefficient was more significant between CRX-527-treated mice and irradiation alone (**Figure 1D**). Meanwhile, we found that the number of LSK (lin^-^sca-1^+^c-kit^+^) cells in CRX-527 mice was greater than that in the irradiated group, which preserved sufficient hematopoietic potential (**Figure 1E**). H&E staining was performed to show the histological changes intuitively, and we found that a more perfect hematopoietic microenvironment, more complete blood sinus structure, and fewer vacuoles and tissue disorders appeared in CRX-527 mice than in irradiated mice. Meanwhile, the number of nucleated cells in the bone marrow of CRX-527 mice was significantly greater than that of mice irradiated alone, which suggested that CRX-527 could effectively alleviate bone marrow injury induced by ionizing radiation (**Figures 1F, G**). In addition, we also found that the density of spleen white pulp decreased significantly after radiation exposure, which indicated that the overall cell density of the spleen decreased greatly after irradiation. However, that was higher in CRX-527 mice, which indicated that CRX-527 could significantly reduce the damage to the spleen induced by ionizing radiation (**Figures 1H, I**). Above all, CRX-527 could protect the structural integrity of the hematopoietic system and create the possibility for the recovery of hematopoietic function after irradiation.

### 3.2 CRX-527 promoted immune-activated differentiation of HSCs

**Figure 2:**
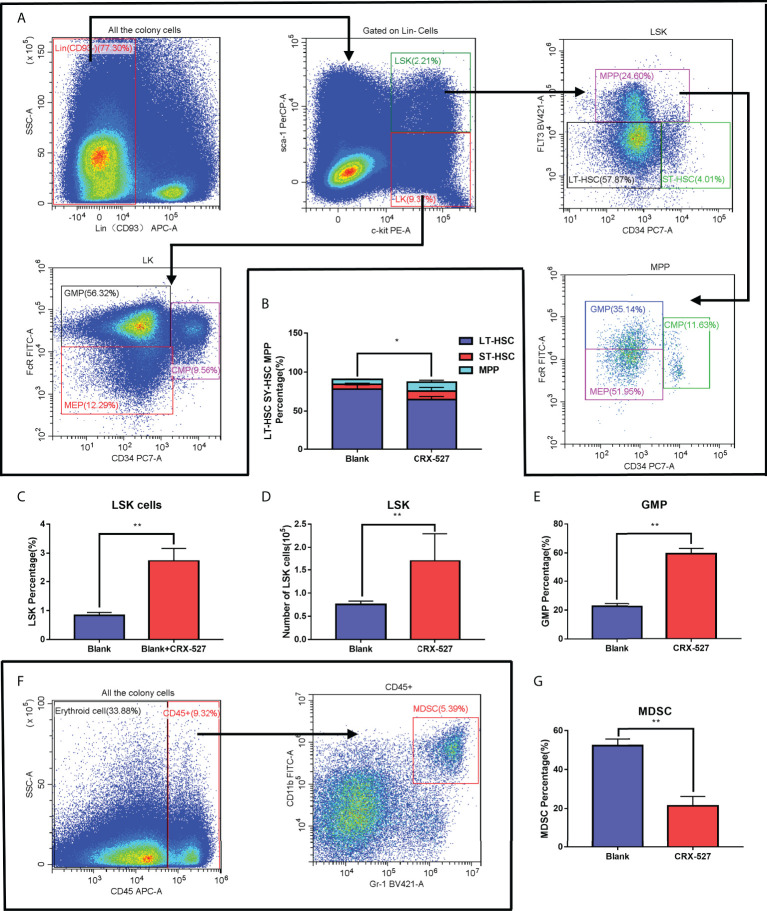
CRX-527 promoted immune-activated differentiation of HSCs. **(A)** The gating pattern of mouse HSC differentiation. **(B)** The proportion of LT-HSCs, ST-HSCs and MPPs with or without CRX-527 treatment. **(C)** The proportion of LSKs with or without CRX-527 treatment. **(D)** The number of LSKs with or without CRX-527 treatment. **(E)** Changes in the proportion of GMPs with or without CRX-527 treatment **(F)** The gating pattern of MDSC differentiation in mice. **(G)** Changes in the MDSC ratio with or without CRX-527 treatment. The data are expressed as the mean ± SEM, *p < 0.05, **p < 0.01.

Flow cytometry was performed to characterize HSCs and precursor cells in bone marrow (**Figure 2A**). CRX-527 maintained a higher number (**Figure 2C**) and proportion (**Figure 2D**) of LSK (Lin^-^sca-1^+^c-kit^+^) cells than normal mice, which confirmed that CRX-527 promoted HSC proliferation. To distinguish the phenotype of HSCs, Flt3 (CD135) and CD34 were labeled based on LSK cells, and we found that the proportion of long-term hematopoietic stem cells (LT-HSCs, CD34^-^CD135^-^LSK) decreased and short-term hematopoietic stem cells (ST-HSCs, CD34^+^CD135^-^LSK) increased after CRX-527 treatment. Meanwhile, the proportion of multipotent hematopoietic progenitors (MPPs, CD34^+^CD135^+^LSK) increased significantly, suggesting that hematopoietic mobilization was activated and that cells differentiated into MPPs (**Figure 2B**). Directional hematopoietic progenitor cells (HPCs), mainly enriched in the Lin^-^cKit^+^Sca-1^-^ (LK) population, were also labeled with FcR and CD34 to distinguish granulocyte/monocyte progenitor cells (GMPs, FcRhighCD34^+^LK), myeloid common precursor cells (CMPs, FcR^+^CD34^+^LK) and lymphoid common precursor cells (CLPs, Sca-1lowIL-7Rαhigh LK), suggesting that HPCs differentiated obviously into GMPs in CRX-527-treated mice (**Figure 2E**). MPPs can also differentiate into GMPs. The proportion of bone marrow-derived inhibitory cells (MDSCs, CD45^+^Gr-1^+^CD11b^+^) in CRX-527-treated mice was lower than that in normal mice (**Figure 2F**), indicating that immunosuppression was relieved and cellular immune excitement was activated (**Figure 2G**). In summary, we found that CRX-527 can stimulate hematopoietic mobilization to immune-activated differentiation and relieve immunosuppression.

### 3.3 Macrophage activation by CRX-527 alleviated IR-induced enterocyte injury

**Figure 3:**
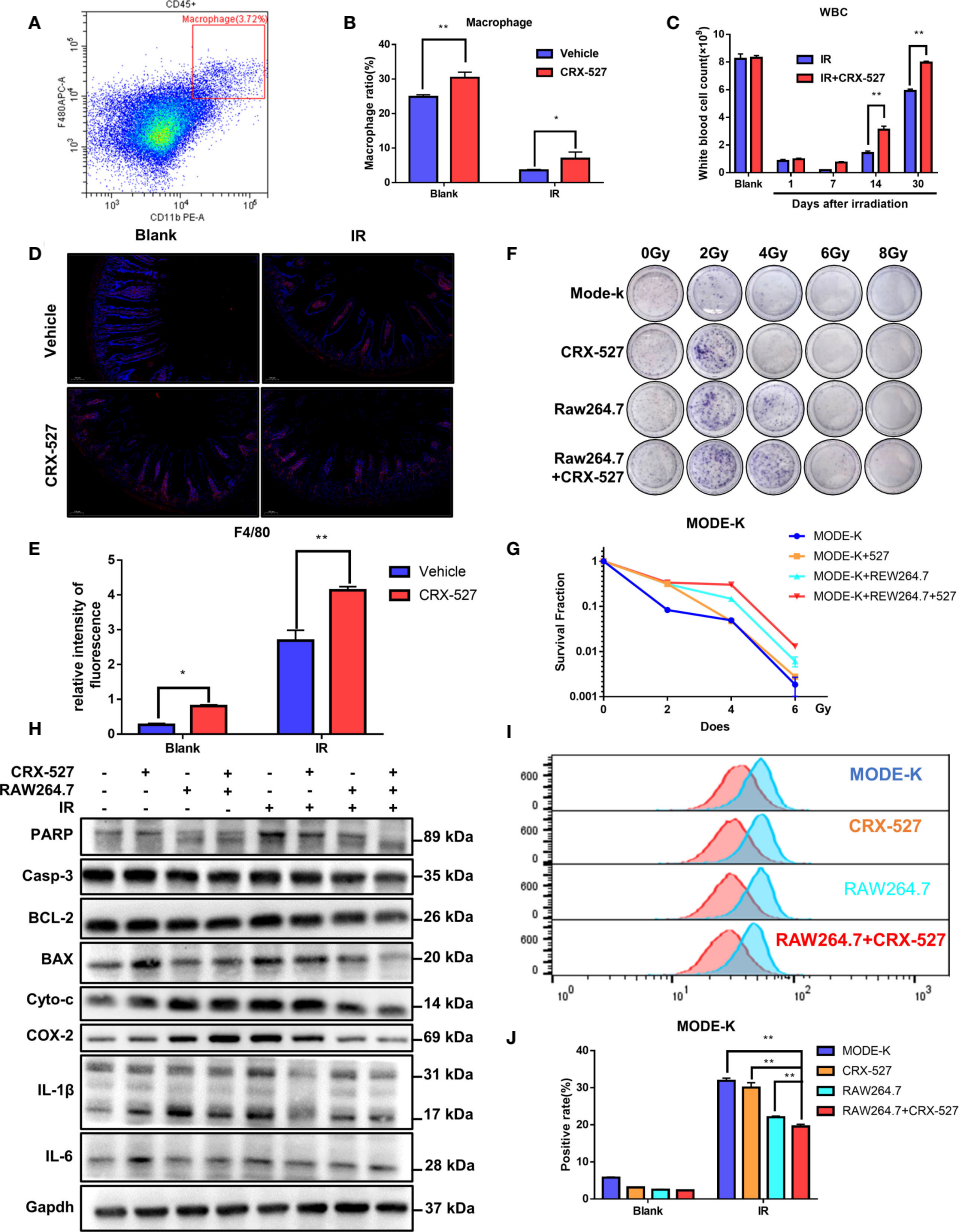
Macrophage activation by CRX-527 alleviated IR-induced enterocyte injury. **(A)** The gating pattern of mouse bone marrow macrophage differentiation. **(B)** After 5 Gy total body irradiation, the proportion of macrophages with or without CRX-527 treatment. **(C)** After 5 Gy total body irradiation, the number of WBCs in peripheral blood with or without CRX-527 treatment. **(D, E)** Intestinal immunofluorescence of F4/80 after 7.5 Gy total body irradiation with or without CRX-527 treatment. **(F)** Clone formation assay of coculture under different irradiation doses. **(G)** Clone formation rate statistics. **(I)** ROS expression in the coculture system of MODE-K after 16 Gy irradiation. **(J)** ROS level statistics. **(H)** Apoptosis- and inflammation-related protein expression in the coculture system was detected under 16 Gy irradiation. The data are expressed as the mean ± SEM, *p < 0.05, **p < 0.01.

The differentiation of HSCs into GMPs benefitted the generation of monocytes and macrophages, and we detected macrophages through flow cytometry labeled with CD11b and F4/80 (**Figure 3A**). The results indicated that the proportion of macrophages (CD11b^+^F4/80^+^) was higher in CRX-527-treated mice both with and without irradiation than in untreated mice (**Figure 3B**). The white blood cells in peripheral blood were significantly higher in CRX-527-treated mice than in irradiated mice throughout the observation period (**Figure 3C**). Moreover, we found that macrophages maintained their high levels in the intestine after CRX-527 treatment (**Figures 3D, E**). CCK8 assay was performed on HIEC and MODE-K cell lines to find the appropriate concentration of CRX-527 (**Figures S2A, B**). Clone formation analysis using the MODE-K-RAW264.7 co-culture system showed that there were obvious differences between the co-cultures with or without RAW264.7 cells (**Figures 3F, G**). Administration of CRX-527 in the co-culture system reduced the protein expression levels of *Caspase-3, Bax, Cytochrome C, Cox-2, Il-1β* and *Il-6* compared with other treatment groups (**Figure 3H**). Furthermore, the CRX-527 co-culture system had less ROS generation and the least suppressed levels of apoptosis and inflammation than any other group after irradiation (**Figures 3I, J**). We also performed clone formation, ROS and Western blotting on HIEC cell line and the results were consistent with the results of MODE-K cell line (**Figures S3A–C**).

### 3.4 CRX-527 promoted the preservation and regeneration of ISCs to protect against IR-induced intestinal injury

**Figure 4:**
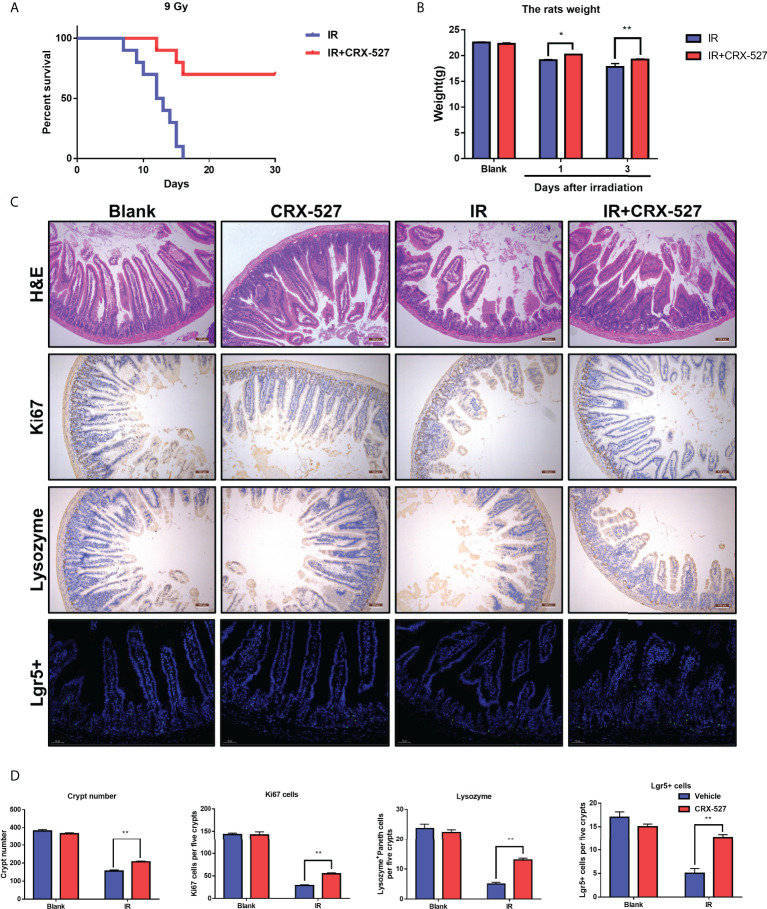
CRX-527 promoted the preservation and regeneration of ISCs to protect against IR-induced intestinal injury. **(A)** Mouse survival curve of 9 Gy total body irradiation. **(B)** Body weight changes in 9 Gy-irradiated mice at different times. **(C)** Intestinal H&E staining in irradiated mice. Intestinal *Ki67* IHC in irradiated mice. Intestinal lysozyme IHC in irradiated mice. Intestinal *Lgr5* FISH in irradiated mice. **(D)** Crypt number statistics. *Ki67* ^+^ cell statistics. *Lysozyme* ^+^ Paneth cell statistics. *Lgr5* ^+^ cell statistics. The data are expressed as the mean ± SEM, *p < 0.05, **p < 0.01.

A series of examinations were performed to evaluate intestinal function. First, a dose of 9 Gy for whole body irradiation was given to monitor survival with or without CRX-527 treatment. Results indicated that CRX-527 increased survival by 80% compared to untreated mice (**Figure 4A**). The changes of the intestines were recorded intuitively (**Figure S4A**), and weight changes were more moderate (**Figure 4B**). Furthermore, H&E staining confirmed that the intestinal villi were ruptured and that the crypt was damaged after irradiation (**Figures 4C, D**). In contrast, CRX-527 preserved the crypt-villi structure well. *Lgr5+* intestinal stem cells (ISCs) are indispensable for intestinal regeneration after irradiation. Compared with the IR group, CRX-527 significantly alleviated the decrease in *Lgr5+* ISCs after irradiation (**Figures 4C, D**). Paneth cells are also known to maintain intestinal homeostasis. There were more *Lysozyme+* Paneth cells reserved after irradiation in CRX-527-treated mice (**Figures 4C, D**). *Villus* protein is involved in the formation of microvilli in the intestinal epithelium. More complete villi were observed and recorded in CRX-527-treated mice than in untreated mice during irradiation (**Figures 4C, D**). Moreover, *Ki67* can act as a marker of epithelial regeneration. Compared with the IR group, the number of *Ki67+* cells in the radiation group decreased significantly, while CRX-527 significantly prevented these changes (**Figures 4C, D**).

### 3.5 CRX-527 protected intestinal homeostasis and function from radiation damage

**Figure 5:**
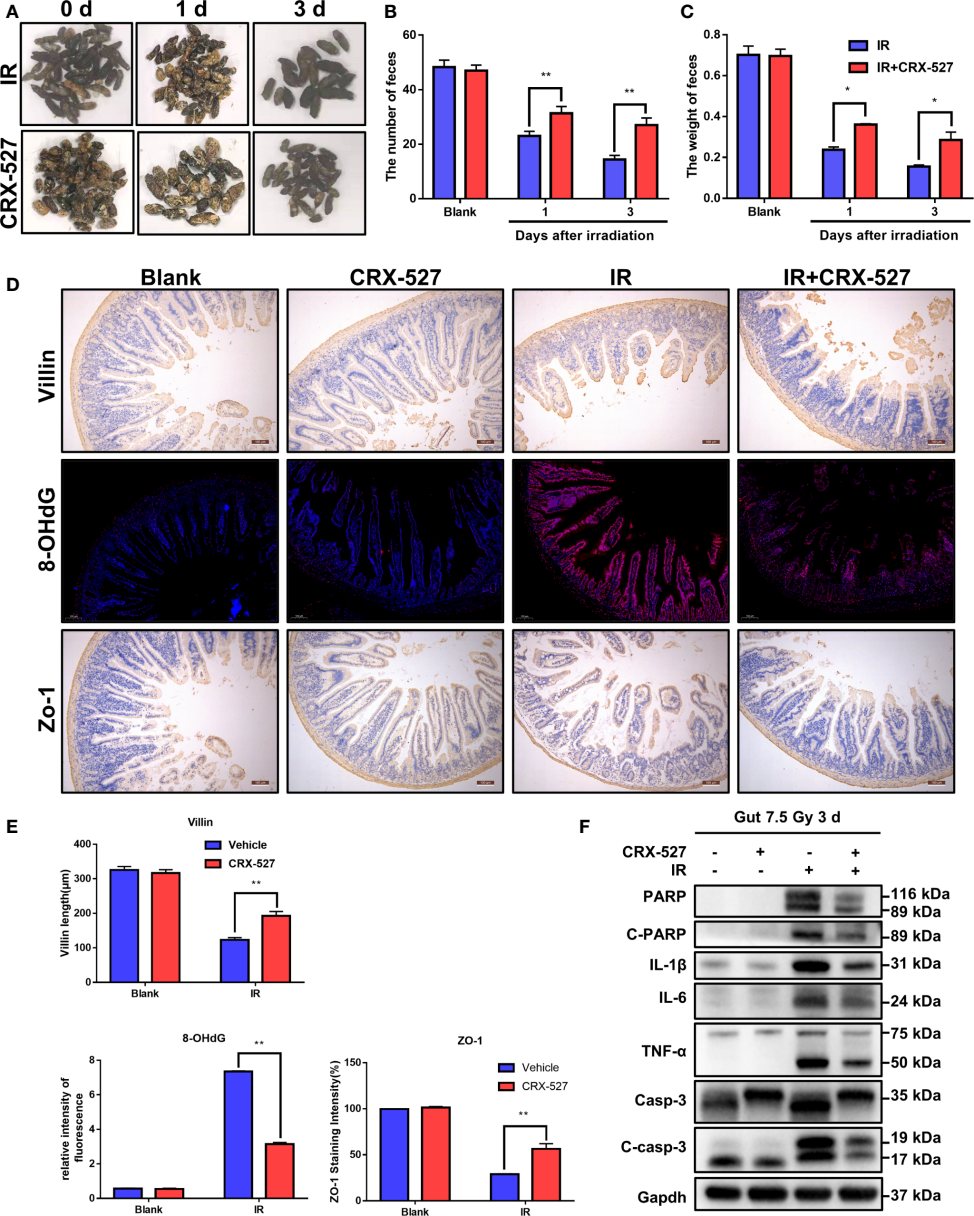
CRX-527 protected intestinal homeostasis and function from radiation damage. **(A)** The morphology of fecal changes in irradiated mice. **(B)** Fecal number statistics. **(C)** Fecal weight statistics. **(D)** Intestinal *Villi* IHC in irradiated mice. Intestinal *8-OHDG* IF in irradiated mice. Intestinal *Zo-1* IHC in irradiated mice. **(E)***Villi* length statistics. *8-OHDG* fluorescence intensity statistics. *Zo-1* ^+^ cell statistics **(F)** The expression of apoptosis- and inflammation-related proteins in the irradiated intestine. The data are expressed as the mean ± SEM, *p < 0.05, **p < 0.01.

We further evaluated the efficiency of CRX-527 on intestinal physiological processes. The feces of mice after irradiation were collected to measure both weight and numbers (**Figure 5A**). Results indicated that CRX-527 mediated better fecal quality, more distinguished quantity, and no watery or loose stools compared to irradiated mice (**Figures 5B, C**). *Zo-1* protein maintains the mechanical barrier and permeability of the intestinal epithelium. We found that more *Zo-1* was retained in CRX-527-treated mice than in irradiated mice, which also verified that CRX-527 could protect intestinal barrier function (**Figures 5D, E**). 8-Hydroxy-2-deoxyguanosine (*8-OHdG*) is considered to be a biomarker of oxidative stress injury. CRX-527 behaved well in suppressing the increase in *8-OHdG* after irradiation (**Figures 5D,E**). Furthermore, Western blot analysis showed that the expression levels of *PARP, IL-1β, IL-6, TNF-α* and *Caspase-3* in the intestinal tissues of CRX-527-treated mice were lower than those in irradiated mice (**Figure 5F**), indicating that CRX-527 could suppress apoptosis of the intestinal epithelium after irradiation.

### 3.6 TLR4 activation mediated the protective effect of CRX-527 on hematopoietic and intestinal radiation injury

**Figure 6:**
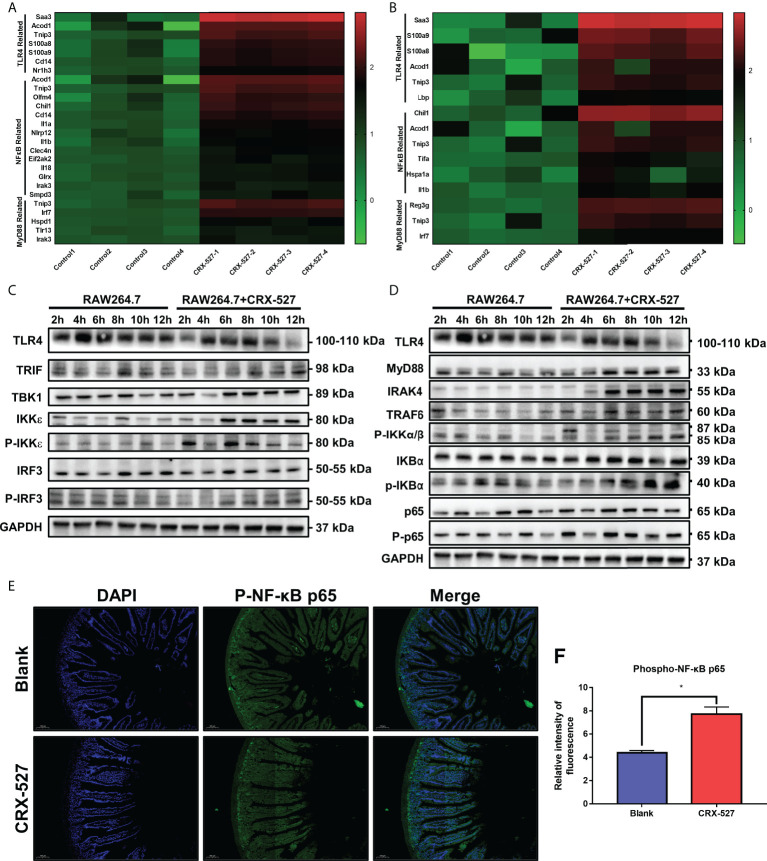
CRX-527 activated TLR4-related pathway in spleen and intestines.Heatmap of TLR4-, MyD88- and NF-κB-related genes in the spleen **(A)** and intestine **(B)** with or without CRX-527 treatment after irradiation. Expression of proteins in the MyD88-dependent pathway **(C)** and MyD88-independent pathway **(D)** in RAW264.7 cells with or without CRX-527 treatment after irradiation. **(E)** Intestinal p-P65 IF in irradiated TLR4^-/-^ mice. **(F)** Fluorescence intensity of p-P65. The data are expressed as the mean ± SEM, *p < 0.05.

We then investigated the mechanism of CRX-527 in hematopoietic and intestinal protection against ionizing radiation. First, transcriptome sequencing was performed to distinguish the different genes from spleens and intestines with or without CRX-527 treatment. The results showed that TLR4-, MyD88- and NF-κB-related genes were significantly increased in CRX-527-treated mice in both spleens (**Figure 6A**) and intestines (**Figure 6B**). To verify the sequencing results, TLR4-related proteins were quantified in RAW264.7 cells, which indicated that following CRX-527 treatment, both the MyD88-dependent classical pathway (**Figure 6C**) and TRIF-dependent nonclassical pathway (**Figure 6D**) were activated downstream of TLR4. We mapped intestinal phospho-NF-κB p65 expression using immunofluorescence (**Figure 6E**) and found that CRX-527-treated mice showed higher levels of phospho-NF-κB p65 expression than normal mice (**Figure 6F**).

**Figure 7:**
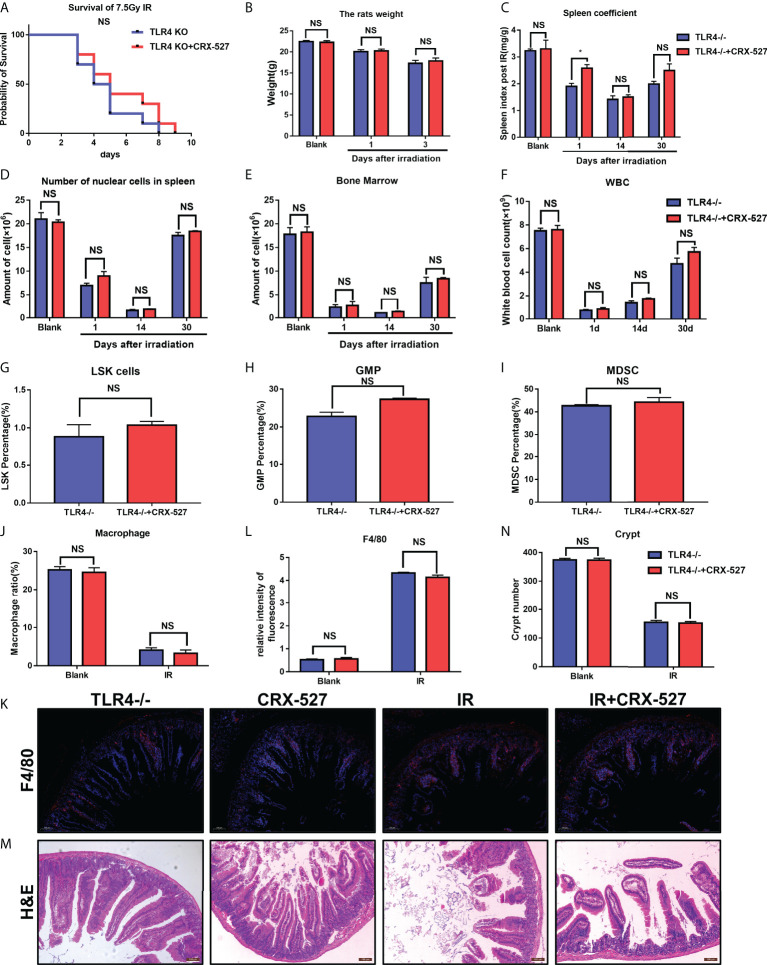
TLR4 activation mediated the protective effect of CRX-527 on hematopoietic and intestinal radiation injury.**(A)** TLR4^-/-^ mice survival curve of 7.5 Gy total body irradiation. **(B)** Weight changes of TLR4^-/-^ mice after irradiation. **(C)** Changes in spleen coefficients in TLR4^-/-^ mice at different times, Effecting of 5 Gy total body irradiation on the hematopoietic system in TLR4^-/-^ mice. Nucleated cell counts in the spleen **(D)** and bone marrow **(E)** of TLR4^-/-^ mice at different times. **(F)** Changes of peripheral leukocytes in TLR4^-/-^ mice. The proportion of LSKs **(G)**, GMPs **(H)**, and MDSCs **(I)** with or without CRX-527 treatment in TLR4^-/-^ mice. **(J)** The proportion of macrophages with or without CRX-527 treatment in irradiated TLR4^-/-^ mice. F4/80 IF **(K)** and statistics **(L)** in TLR4^-/-^ mice **(M)** Intestinal H&E staining of TLR4^-/-^ mice **(N)** and intestinal crypt counts. The data are expressed as the mean ± SEM, *p < 0.05. NS, no significance.

Homozygous TLR4 knockout mice (TLR4^-/-^) were used to further verify the efficiency of CRX-527 *in vivo*. It was found that CRX-527 had no significant effect on survival rate (**Figure 7A**), body weight (**Figure 7B**), spleen coefficient (**Figure 7C**), number of nucleated cells in spleen (**Figure 7D**), number of nucleated cells (**Figure 7E**) in bone marrow and peripheral blood (**Figure 7F**) of irradiated knockout mice. Meanwhile, there were no significant differences in hematopoietic mobilization and differentiation, particularly with regard to LSKs (**Figure 7G**), GMPs (**Figure 7H**), MDSCs (**Figure 7I**) and macrophages (**Figure 7J**). Beyond that, there was no significant difference in F4/80 between with and without CRX-527 treatment in TLR4^-/-^ mice (**Figures 7K, L**), indicating that no increase in macrophages was activated in TLR4^-/-^ mice. No significant difference was found in the number of crypts by H&E staining (**Figures 7M, N**). Intestinal tract of TLR4^-/-^ mice was also tested for *Ki67, 8-OHDG, Lysozyme* and *Villin*, and no significant difference was found between the TLR4^-/-^ group and the CRX-527 group (**Figures S5A, B**).

## 4 Discussion

Ionizing radiation can cause acute radiation syndrome, resulting in hematopoietic depletion, gastrointestinal collapse and cerebrovascular injury (13). The hematopoietic system is highly sensitive to radiation. Even low-dose irradiation can cause significant changes and dysfunction in hematopoiesis (14). In the pathophysiological process of acute radiation sickness, changes in hematopoietic organs appear earlier, mainly manifested as the reduction and injury of nucleated cells in peripheral blood and bone marrow, hematopoietic dysfunction and even failure, and eventually lead to anemia, hemorrhage, infection, metabolic disorder and death (15). Hematopoietic radiation injury is obviously time-sensitive and can be used as the basis for classification, diagnosis and prognosis (16). At present, there is still no research on the radioprotective effects of CRX-527, let alone their effects on the hematopoietic system. In this study, we verified the effect of CRX-527 on the hematopoietic system, determined the phenotypic differentiation of HSCs, and revealed an excitatory effect on the immune system (17, 18). On this basis, we observed changes in intestinal structure and function in mice after ionizing radiation and revealed the radioprotective mechanism of CRX-527 in TLR4-related pathway activation. This research is a pioneering work.

Irradiation can reduce the survival rate of mice by 30% (19). In this study, mice were irradiated, the average survival time of mice treated with CRX-527 was significantly prolonged, and the survival rate was significantly improved. Injury to tissues and organs related to the hematopoietic system, including bone marrow, spleen and peripheral blood, was relieved. Mouse HSCs account for only 1% of bone marrow cells enriched in the LSK population (20). HPCs are mainly enriched in the LK population. IR can cause a decrease in the number and proportion of LSK, while CRX-527 could alleviate it, which could provide necessary guarantees for the self-renewal and peripheral differentiation of hematopoietic stem cells after ionizing radiation. Based on the strong heterogeneity of the LSK cell population, it can be divided into LT-HSCs, ST-HSCs, and MPPs. LT-HSCs are the most primitive HSCs, which can differentiate into ST-HSCs and MPPs and then differentiate into various cells of the whole blood system. CRX-527 can increase the ratio of MPPs and ST-HSCs in mice, while the ratio of LT-HSCs decreased slightly, which indicated that CRX-527 could promote the differentiation of LT-HSCs into MPPs, thus allowing MPPs to differentiate into macrophages (21).

MPPs can further differentiate into CMPs and CLPs that can continue to differentiate into precursor cells of various lymphocytes and finally differentiate into T cells, B cells, DC cells and NK cells. On the other hand, CMPs can continue to differentiate into GMPs and MEPs. GMPs can further differentiate into monocyte macrophages and granulocytes (22).

HPCs, similar to MPPs, are the direct source of peripheral blood granulocytes and are sensitive to radiation. At present, the confirmed hematopoietic progenitor cells are as follows: ① Erythrocyte hematopoietic progenitor cells can form red blood cell colonies, also known as red blood cell colony forming units (CFU-Es), under the action of erythropoietin (EPO) (23). ① Megakaryocyte line hematopoietic progenitor cells can form megakaryocyte colonies, also known as megakaryocyte line colony forming units (CFU-Ms), under the action of thrombopoietin (24). ③ Neutrophil-macrophage hematopoietic progenitor cells can form neutrophil-macrophage colonies, also known as granulocyte-macrophage colony forming units (CFU-GMs), under the action of granulopoietin (generated by macrophages) (25). CRX-527 can promote the directional differentiation of HPCs into GMPs, which was consistent with the direct effect on MPP and guaranteed the proliferation of granulocytes and macrophages.

Myeloid-derived suppressor cells (MDSCs) are a group of heterogeneous cells derived from bone marrow (26). As the precursor of dendritic cells (DCs), macrophages and/or granulocytes, MDSCs have the ability to significantly inhibit the immune cell response and are derived from bone marrow progenitor cells and immature myeloid cells (IMCs) (27). Under normal circumstances, precursors of DCs, macrophages and granulocytes can quickly differentiate into mature DCs, macrophages and granulocytes and enter corresponding organs and tissues to exhibit normal immune function, in which IMCs account for approximately 0.5% of peripheral blood mononuclear cells. Under pathological conditions such as tumors, infection, inflammation, septicemia, and surgical injury, the maturation of myeloid-derived precursor cells is blocked due to the action of cytokines, thus they remain at various stages of differentiation and become MDSCs with immunosuppressive functions. In previous studies, TLR ligands have been shown to activate many different signaling pathways involved in MDSCs, including STAT6, STAT1 and NF-κB. Notably, CRX-527 can improve the immune response of macrophages and granulocytes by reducing the proportion of MDSCs and the suppression ability of immune cells (28).

Macrophages and monocytes are both phagocytes that participate in nonspecific defense (innate immunity) and specific defense (adaptive immunity) in vertebrates, in which they phage fragments and pathogens in fixed or dissociated cells and activate lymphocytes or other immune cells to make them respond to pathogens (29). We found that CRX-527 could stimulate mouse bone marrow to generate a large number of macrophages (CD11b^+^F4/80^+^) for immune defense. In addition, the changes in peripheral blood white blood cells (including monocytes, macrophages, neutrophils, DCs and NK cells) are consistent with the clinical stage of radiation sickness in the time phase, so they are often used as one of the criteria for judging the clinical course, condition and prognosis of radiation sickness (30). CRX-527 alleviated the decrease in leukocyte levels induced by ionizing radiation, mainly by increasing the proportion and number of macrophages in peripheral blood leukocytes. Above all, CRX-527 could maintain the proportion and quantity of LSK, promote the mobilization of HSCs and induce them to differentiate into MPPs-GMPs-macrophages.

The intestines have the largest macrophage pool in the body, where macrophages mainly come from intestinal local macrophages and bone marrow HSCs (6). According to previous research reports, the level of bone marrow-derived macrophages is associated with the complexity of the intestinal flora and mediated by the existing TLR ligands in the serum, which can maintain intestinal mucosal homeostasis, promote intestinal epithelium and crypt regeneration, and regulate positive immune function in the intestine (5). For example, in the process of senescent cell clearance and tissue remodeling, intestinal macrophages can produce a variety of cytokines, which can stimulate the proliferation of epithelial progenitor cells in the intestinal crypts, regulate the integrity of the epithelial barrier, and maintain intestinal homeostasis (31). TLR4 was highly expressed on the surface of macrophages, and it was also expressed in small intestinal epithelial cells, but at a much lower level than that in macrophages (32). We suspected that CRX-527 could not only protect the intestine from radiation damage through the TLR4 pathway but also activate the immune response through macrophages to achieve more effective protection of the intestine. To verify this conjecture, THP-1-HIEC and RAW264.7-MODE-K co-culture systems were used to simulate the therapeutic effect of macrophages on small intestinal epithelial cells (33), in which CRX-527 behaved well and far exceeded the protective effect of CRX-527 on small intestinal epithelial cells alone, indicating that CRX-527 can activate the TLR4 pathway of macrophages to protect small intestinal epithelial cells from IR-induced damage. In addition, in the co-culture system, CRX-527 also suppressed the generation of ROS, the activation of apoptosis and the release of inflammatory factors in small intestinal epithelial cells.

Studies have shown that ISCs, intestinal epithelial cells, goblet cells, Pan’s cells, and some undifferentiated cells have strong sensitivity to irradiation (4). A certain dose of radiation can cause extensive damage and destruction, eventually leading to the occurrence of intestinal radiation sickness, which is mainly manifested as intestinal mucosal injury and shedding, loss of crypt proliferation ability, villus rupture and peeling, loss of intestinal shielding function, increase of intestinal permeability, and immune deficiency (34). Patients may show acute symptoms characterized by gastrointestinal symptoms such as vomiting, diarrhea and bloody stool. At present, the recovery rate of intestinal radiation sickness is extremely low clinically, so more research on the molecular mechanism and treatment strategies of intestinal radiation sickness is urgently needed (35).

Crypt basal ISCs, specially labeled with *Lgr5*, can generate differentiated intestinal cells, which are necessary to maintain balance *in vivo* and to regenerate the intestinal crypt-villus structure during irradiation (36). CRX-527 can preserve the normal crypt-villus structure of mouse small intestinal tissue after ionizing radiation, which was attributed to the constant level of *Lgr5^+^* cells, and reserve the regeneration ability of intestinal cells, including Paneth cells (*Lysozyme^+^*), intestinal epithelial cells (*Villin^+^*) and transient expansion cells (*Ki67^+^*). In addition, CRX527 can also maintain intestinal barrier function and homeostasis. Moreover, CRX-527 protected intestinal tissue by suppressing apoptosis and releasing inflammatory factors (4).

Hoffmann JA first discovered TLR molecules in Drosophila in 1996 (37). Subsequently, Burdelya LG reported the effect of TLR5 in radiation prevention in 2008. Since then, the role of TLRs in radiation injury prevention has attracted increasing attention. As a member of the TLR family, TLR4 plays an important role in innate immunity (38). In previous reports, TLR4 also participated in radiation protection, and the survival rate of irradiated mice was significantly improved after TLR4 was activated by LPS. However, as a proinflammatory agent, LPS itself has inevitable toxicity, so it is crucial that we find a series of radiation protection agents with high efficiency and low toxicity. To date, CRX-527 has been found to be an effective agonist of the TLR4 signaling pathway (39). After TLR4 dimerization, IRAK1 is phosphorylated by IL-1 receptor-associated kinase (IRAK) *via* IRAK4 in myD88-dependent pathways (40). Phosphorylated IRAK1 activates tumor necrosis factor receptor-associated Factor 6 (TRAF6) (41), leading to the activation of transforming growth factor-β-activated protein kinase 1 (TAK1), which binds TGF-β-activated kinase 1/MAP3K7-binding proteins 1 (TAB1), TAB2, and TAB3. The TAK1/TAB1/TAB2/TAB3 complex then phosphorylates the IKK complex, which has two catalytic subunits, IKKα and IKKβ and IKKγ/NEMO (42). This is followed by the degradation of the NFκB inhibitor (IκB), which leads to the development of the NF-κB dimer and transfer to the nucleus. For the alternative TRIF pathway, cytoplasmic TRIF-associated adapter molecule (TRAM) induces the recruitment of interferon-β (TRIF) to the TLR4 receptor by an adaptor containing the TIR domain. CRX-527-induced activation of the TLR4 pathway leads to internalization of the TLR4/TRAM/TRIF complex through endosomes. RIP1 signals TRAF6, which results in TNF-R-associated Factor 3 (TRAF3) recruitment to TRIF, thus initiating the activation of IKKϵ and tank-binding kinase 1 (TBK1) (43). Interferon regulatory Factor 3 (IRF3) is then transferred to the nucleus, resulting in the generation of inflammatory mediators and type 1 interferon. TLR4/MD-2 complexes expressed on the surface of macrophages, monocytes and dendritic cells sense micromolar concentrations of TLR4 ligand and trigger the generation of various pro-inflammatory mediators, which provides the basis for the TLR4 ligand CRX-527 to reduce intestinal injury after irradiation by activating macrophages.

## 5 Conclusion

In this study, we demonstrated that CRX-527 protected against irradiation-induced hematopoietic and intestinal injury. Compared with LPS, CRX-527 was less toxic *in vivo* and *in vitro*. Mechanistically, the stimulation of CRX-527 significantly increased the proportion and number of HSCs and promoted their differentiation into macrophages, activating immune defense. On this basis, we observed positive changes in intestinal structure and function in mice after ionizing radiation. Activation of the TLR4-related pathway mediated the protective effect of CRX-527 on hematopoietic and intestinal radiation injury.

## Data availability statement

The data presented in the study are deposited in the Sequence Read Archive (SRA) repository, accession number PRJNA872220.

## Ethics statement

The animal study was reviewed and approved by PLA Rocket Force Characteristic Medical Center.

## Author contributions

DL, SD, and CL contributed equally to this work. DL carried out all biology experiments. SD designed experiments. CL wrote the manuscript. JD, ZC, HY, WL, SW, RP, MZ, and QJ provided help with the experiment. FL and RZ provided guidance for the experiment. All authors contributed to the article and approved the submitted version.

## Funding

This work was supported by Military Research Program (20QNPY063) and 389 National Natural Science Foundation of China (82003388, 81872559).

## Acknowledgments

Research group members 390 Xiaoli Lv, Jing Ma, Shuhan Jia, and Ziqing Zhang also made contributions to this work.

## Conflict of interest

The authors declare that the research was conducted in the absence of any commercial or financial relationships that could be construed as a potential conflict of interest.

## Publisher’s note

All claims expressed in this article are solely those of the authors and do not necessarily represent those of their affiliated organizations, or those of the publisher, the editors and the reviewers. Any product that may be evaluated in this article, or claim that may be made by its manufacturer, is not guaranteed or endorsed by the publisher.

## Supplementary material

Supplementary Figure 1
**(A)** Comparison of toxicity between CRX-527 and LPS at same dose in different concentrations. **(B)** Spleen changes in CRX-527 treated mice and other groups after 5 Gy irradiation. **(C)** Changes of bone marrow CD3^+^ ratio between CRX-527 treated mice and other groups after 5 Gy irradiation. **(D)** Change statistics of CD3^+^ ratio. The data are expressed as the mean ± SEM, *p < 0.05, **p < 0.01.

Supplementary Figure 2
**(A)** Optimum concentration screening of CRX-527 for HIEC and MODE-K cell lines. **(B)** Optimum dosing concentration of CRX-527 for HIEC at different irradiation doses. The data are expressed as the mean ± SEM, *p < 0.05, **p < 0.01.

Supplementary Figure 3
**(A)** Effects of different doses of irradiation on HIEC clone formation. **(B)** Effects of CRX-527 on ROS in HIEC-THP-1 co-culture system after irradiation. **(C)** Effects of CRX-527 on apoptosis and inflammation related protein expression in HIEC-THP-1 co-culture system after irradiation. The data are expressed as the mean ± SEM, *p < 0.05, **p < 0.01.

Supplementary Figure 4 Effects of 7.5 Gy irradiation on intestine of mice.

Supplementary Figure 5
**(A)** Intestinal *Ki67*, *Lysozyme*, *Villin* immunohistochemistry, *8-OHDG* immunofluorescence in TLR4-/- mice; **(B)** Intestinal *Ki67*, *Lysozyme*, *Villin*, *8-OHDG* statistics in TLR4-/- mice. The data are expressed as the mean ± SEM, *p < 0.05, **p < 0.01.
