# Supplementary figures and images for: CRX-527 induced differentiation of HSCs protecting the intestinal epithelium from radiation damage
Source: Front Immunol. 2022 Aug 30;13:927213. doi: 10.3389/fimmu.2022.927213 (PMC9468934; doi:10.3389/fimmu.2022.927213)

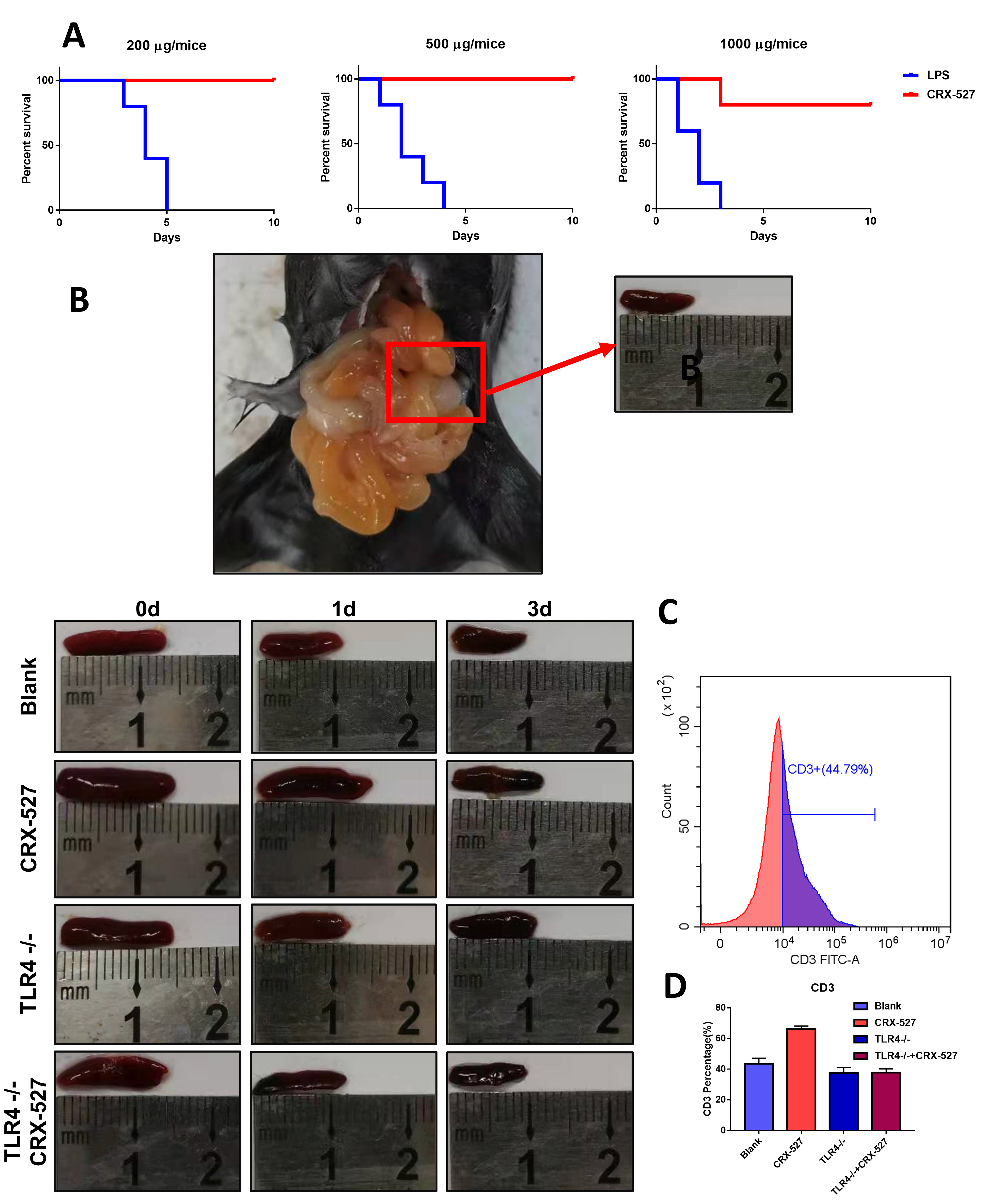

Supplement: Supplementary Figure 1 — (A) Comparison of toxicity between CRX-527 and LPS at same dose in different concentrations. (B) Spleen changes in CRX-527 treated mice and other groups after 5 Gy irradiation. (C) Changes of bone marrow CD3+ ratio between CRX-527 treated mice and other groups after 5 Gy irradiation. (D) Change statistics of CD3+ ratio. The data are expressed as the mean ± SEM, *p < 0.05, **p < 0.01. [file Image_1.tif]

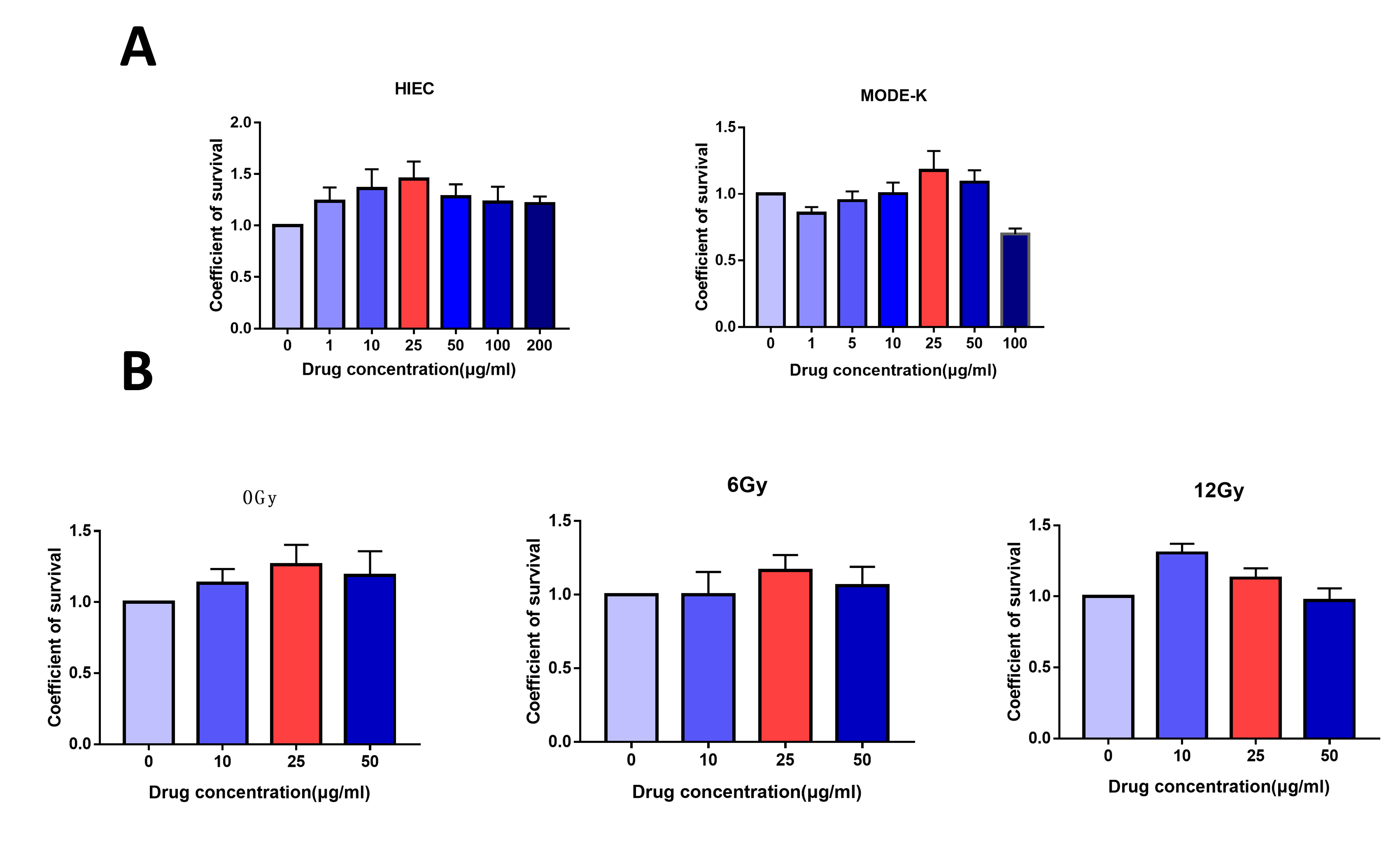

Supplement: Supplementary Figure 2 — (A) Optimum concentration screening of CRX-527 for HIEC and MODE-K cell lines. (B) Optimum dosing concentration of CRX-527 for HIEC at different irradiation doses. The data are expressed as the mean ± SEM, *p < 0.05, **p < 0.01. [file Image_2.tif]

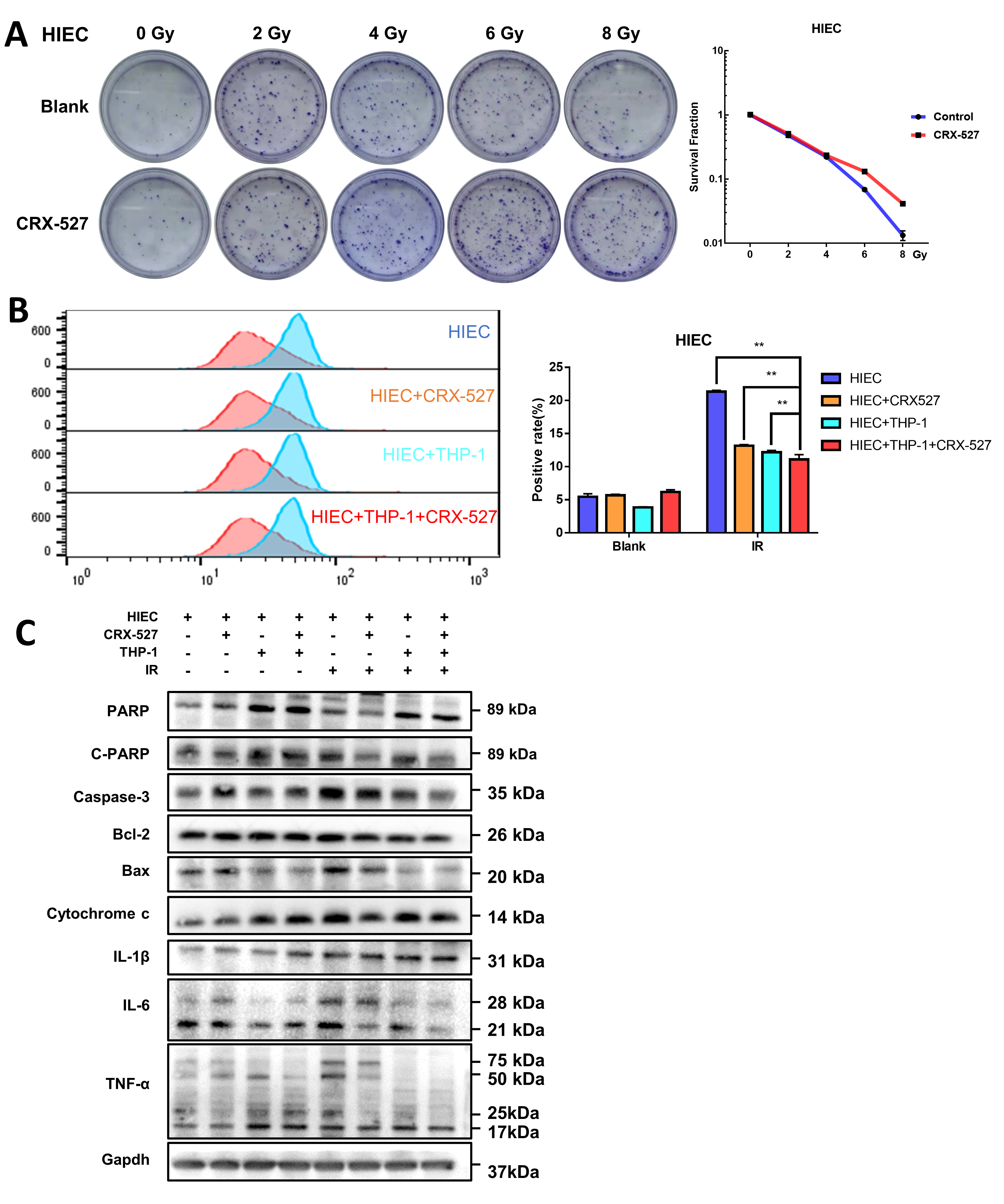

Supplement: Supplementary Figure 3 — (A) Effects of different doses of irradiation on HIEC clone formation. (B) Effects of CRX-527 on ROS in HIEC-THP-1 co-culture system after irradiation. (C) Effects of CRX-527 on apoptosis and inflammation related protein expression in HIEC-THP-1 co-culture system after irradiation. The data are expressed as the mean ± SEM, *p < 0.05, **p < 0.01. [file Image_3.tif]

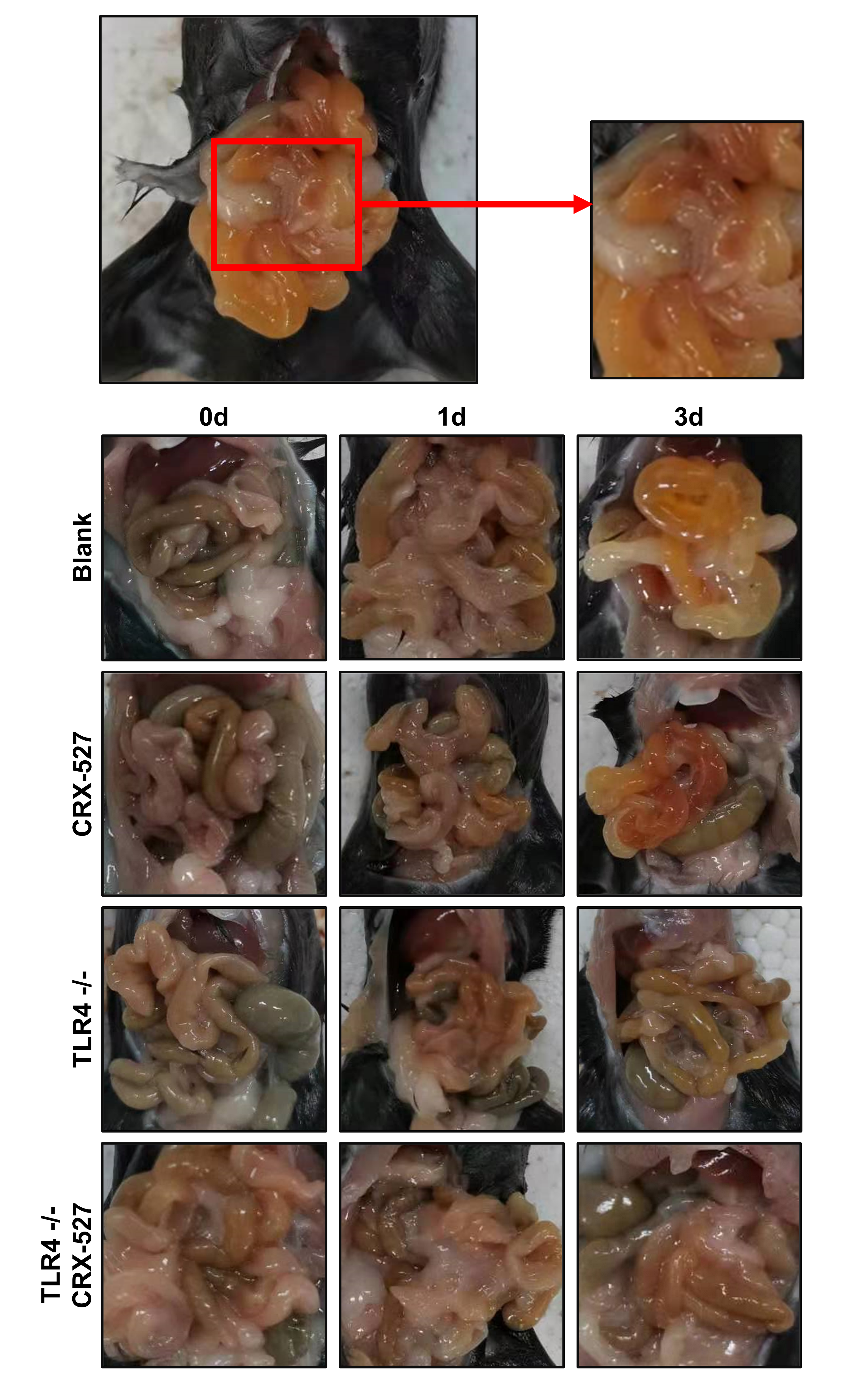

Supplement: Supplementary Figure 4 — Effects of 7.5 Gy irradiation on intestine of mice. [file Image_4.tif]

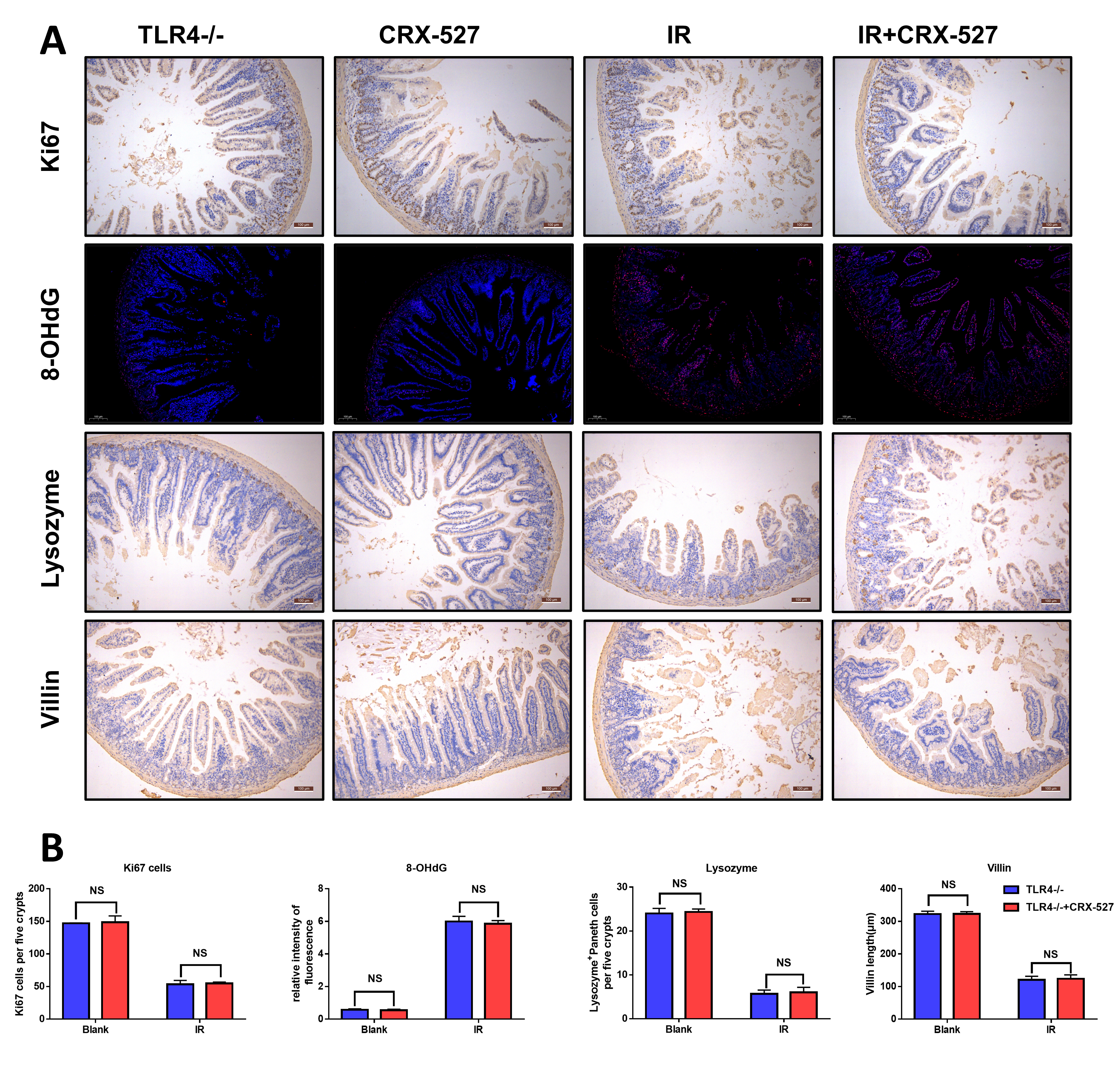

Supplement: Supplementary Figure 5 — (A) Intestinal Ki67, Lysozyme, Villin immunohistochemistry, 8-OHDG immunofluorescence in TLR4-/- mice; (B) Intestinal Ki67, Lysozyme, Villin, 8-OHDG statistics in TLR4-/- mice. The data are expressed as the mean ± SEM, *p < 0.05, **p < 0.01. [file Image_5.tif]
